# DNS dataset for malicious domains detection

**DOI:** 10.1016/j.dib.2021.107342

**Published:** 2021-09-04

**Authors:** Cláudio Marques, Silvestre Malta, João Paulo Magalhães

**Affiliations:** a Escola Superior de Tecnologia e Gestão, Politécnico de Viana do Castelo, Viana do Castelo 4900-348, Portugal; b ADiT-Lab, Escola Superior de Tecnologia e Gestão, Politécnico de Viana do Castelo, Viana do Castelo 4900-348, Portugal; c CIICESI, Escola Superior de Tecnologia e Gestão, Politécnico do Porto, Felgueiras, Portugal

**Keywords:** DNS, Firewall, Machine learning, Cybersecurity

## Abstract

The Domain Name Service (DNS) is a central point in the functioning of the internet. Just as organizations use domain names to enable the access to their computational services, malicious actors make use of domain names to point to the services under their control. Distinguishing between non-malicious and malicious domain names is extremely important, as it allows to grant or block the access to external services, maximizing the security of the organization and users. Nowadays there are many DNS firewall solutions. Most of these are based on known malicious domain lists that are being constantly updated. However, in this way, it is only possible to block known malicious communications, leaving out many others that can be malicious but are not known. Adopting machine learning to classify domains contributes to the detection of domains that are not yet on the block list. The dataset described in this manuscript is meant for supervised machine learning-based analysis of malicious and non-malicious domain names. The dataset was created from scratch, using publicly DNS logs of both malicious and non-malicious domain names. Using the domain name as input, 34 features were obtained. Features like the domain name entropy, number of strange characters and domain name length were obtained directly from the domain name. Other features like, domain name creation date, Internet Protocol (IP), open ports, geolocation were obtained from data enrichment processes (e.g. Open Source Intelligence (OSINT)). The class was determined considering the data source (malicious DNS log files and non-malicious DNS log files). The dataset consists of data from approximately 90000 domain names and it is balanced between 50% non-malicious and 50% of malicious domain names.

## Specifications Table

**Table:** 

Subject	Artificial Intelligence
Specific subject area	Machine Learning
Type of data	Dataset
	Table
	Figure
	Graph
	Python code
How data were acquired	DNS logs, well known malicious domain lists, OSINT sources
Data format	Analyzed
	Raw
	Analyzed
	Filtered
Parameters for data collection	The data collection for non-malicious domains were based on DNS logs and using four types of records (A, AAAA, CNAME and MX). The malicious domains were based on a well-known suspicious domain lists.
Description of data collection	The non-malicious domains were collected from Rapid7 Labs [1] that provides datasets of DNS requests from their Project Sonar. This data is open and is provided a structured schema that allows a simpler extraction process. 45000 domains were randomly selected from these lists. The malicious domains were collected from the SANS Internet Storm Center (SANS) [2] public list. Are well-known suspicious domains and each of them is reported to common virus detectors publicly available. 45000 domains were randomly selected from the list.
Data source location	Data was gathered between September 2020 and November 2020 using the Rapid7 Labs [1] open data repository and from a well-known malicious list provided by SANS [2].
Data accessibility	The data is hosted in a public repository.
	Repository name: Mendeley Data
	Data identification number: https://doi.org/10.17632/623sshkdrz.5
	Direct URL to data: https://doi.org/10.17632/623sshkdrz.5

## Value of the Data

• Considering the number of malicious domains registered every day where some of them are only active for short periods of time, there it is of extreme importance to automate the detection of these malicious domains in a timely manner. Develop and apply Machine Learning (ML) algorithms for this purpose it is very promising;
• The data could be used and applied by the scientific community to study and improve the accuracy of detection of malicious domains names in a timely manner towards the development of a real-time DNS firewall;
• The data provides the identification and valuation of two classes of domains, malicious and non-malicious, a topic valuable to computer and data science investigations;
• The data shows patterns to each class giving the possibility to compare and analyze it in a multi-feature study;
• Contribution to the lack of datasets on malicious and non-malicious domains based on DNS logs which are especially important to the field.

## 1 Data Description

The data consists of a Comma-separated Values (CSV) file providing thirty-four features for each DNS domain. The features are presented in Table 1, containing the description, the data type and its default value. The decimal values are rounded to the first decimal place. The X denotes the type of data and N/A refers to the non-applicable field. The domain feature was encoded to anonymize the real DNS domain name. The DNSRecordType feature was left in the dataset for filtering purposes, allowing the analyst to select data according the DNS record type (A, AAAA, CNAME and MX).The enumerated data types are described in Table 2.

**Table 1:** Dataset features with description, data types and default value.

		Data Type					
Feature	Description	Text	Boolean	Integer	Decimal	Enumerate	Default Value
Domain	Baseline DNS used to enrich data (derive features)	X					N/A
DNSRecordType	DNS record type queried	X					N/A
MXDnsResponse	The response from a DNS request for the record type MX		X				False
TXTDnsResponse	The response from a DNS request for the record type TXT		X				False
HasSPFInfo	If the DNS response has Sender Policy Framework attribute		X				False
HasDkimInfo	If the DNS response has Domain Keys Identified Email attribute		X				False
HasDmarcInfo	If the DNS response has Domain-Based Message Authentication		X				False
IP	The IP for the domain	X					null
DomainInAlexaDB	If the domain it’s registered in the Alexa DB		X				False
CommonPorts	If the domain it’s available for common ports (80, 443, 21, 22, 23, 25, 53, 110, 143, 161, 445, 465, 587, 993, 995, 3306, 3389, 7547, 8080, 8888)		X				False
CountryCode	The country code associated with the IP of the domain	X					null
RegisteredCountryCode	The country code defined in the domain registration process (WHOIS)	X					null
CreationDate	The creation date of the domain (WHOIS)					X	0
LastUpdateDate	The last update date of the domain (WHOIS)					X	0
ASN	The Autonomous System Number for the domain			X			-1
HttpResponseCode	The HTTP/HTTPS response code for the domain					X	0
RegisteredOrg	The organization name associated with the domain (WHOIS)	X					null
SubdomainNumber	The number of sub-domains for the domain			X			0
Entropy	The Shannon Entropy of the domain name			X			0
EntropyOfSubDomains	The mean value of the entropy for the sub-domains			X			0
StrangeCharacters	The number of characters different from [a-zA-Z] and considering the existence maximum of two numeric integer values			X			0
TLD	The Top Level Domain for the domain	X					null
IpReputation	The result of the blocklisted search for the IP		X				False
DomainReputation	The result of the blocklisted search for the domain		X				False
ConsoantRatio	The ratio of consonant characters in the domain				X		0
NumericRatio	The ratio of numeric characters in the domain				X		0
SpecialCharRatio	The ratio of special characters in the domain				X		0
VowelRatio	The ratio of vowel characters in the domain				X		0
ConsoantSequence	The maximum number of consecutive consonants in the domain			X			0
VowelSequence	The maximum number of consecutive vowels in the domain			X			0
NumericSequence	The maximum number of consecutive numerics in the domain			X			0
SpecialCharSequence	The maximum number of consecutive special characters in the domain			X			0
DomainLength	The length of the domain			X			N/A
Class	The class of the domain (malicious = 0 and non-malicious = 1)			X			N/A

**Table 2:** Values description for enumeration features where X denotes all possible values.

Feature	Values description
	Without data = 0
CreationDate	Until one month = 1
	Until six months = 2
LastUpdateDate	Until one year = 3
	After one year = 4
HttpResponseCode	Without data = 0
	1XX response = 1
	2XX response = 2
	3XX response = 3
	4XX response = 4
	5XX response = 5

The enumerations data type identified in Table 1 are presented in Table 2. The enumerations have been created to prepare the data to be better supported by the ML algorithms. The values adopted resulted from the analysis of studies, like [3], [4], [5] that focus on domains names gathering and DNS features to improve the malicious detection based on ML applications.

**Fig. 1:**
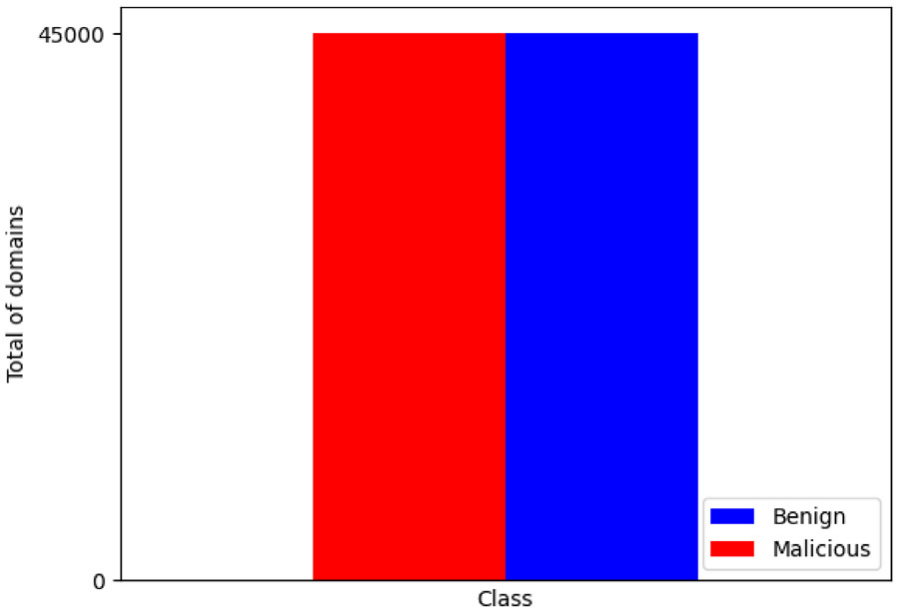
Class label distribution graph.

The dataset is targeted for Supervised ML Classification. It is a binary classification predictive modeling, since according the features presented in Table 1, the class is zero (0) for malicious domains and one (1) for non-malicious domains. The dataset is also balanced and distributed as illustrated in Fig. 1. The ML classification should consider the default dataset characteristics or adapt the dataset for other types of data analysis.

The total number of complete entries without “null” values in the dataset is 11547 (12,83%). There exist 78453 (87,17%) rows where at least one of the features it is “null”. The “null” values should be considered in the data preparation phase allowing the researcher to choose the best approach to handle the “null” values.

**Fig. 2:**
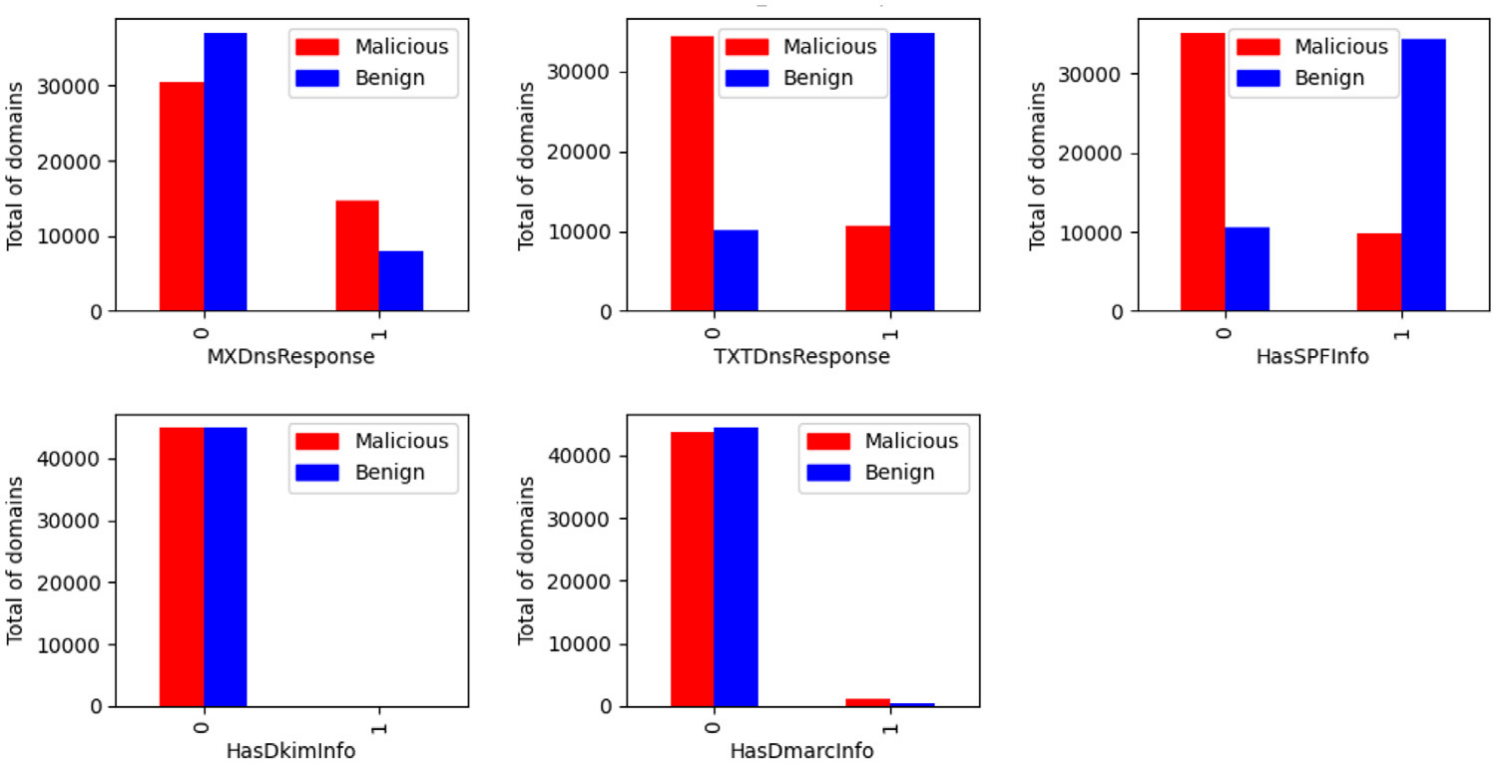
DNS response by class.

The features related to the DNS response are illustrated in the Fig. 2. The figure shows the type of DNS record type requested per class.

**Fig. 3:**
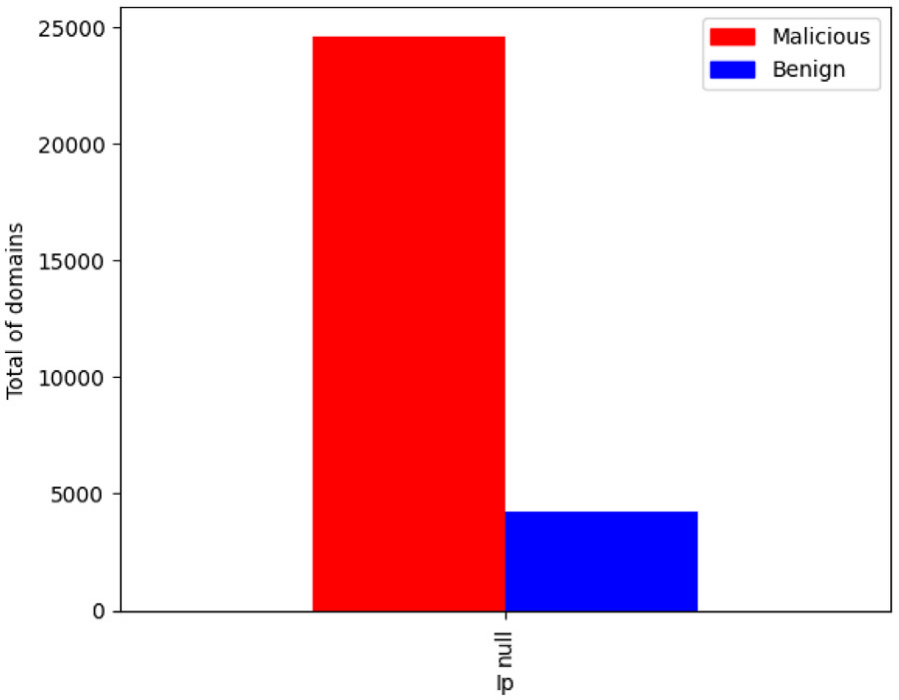
IP null values per class.

As expected the feature IP has a large number of different values. In Fig. 3 is presented a correlation between “null” IP address values per class label. The figure shows that there is a high number of malicious domains that do not have an associated IP. This may indicate that the IP has not yet been assigned to the domain or that the domain has already been used for cyberattacks and its DNS mapping has been removed. This analysis is useful for posterior feature selection.

**Fig. 4:**
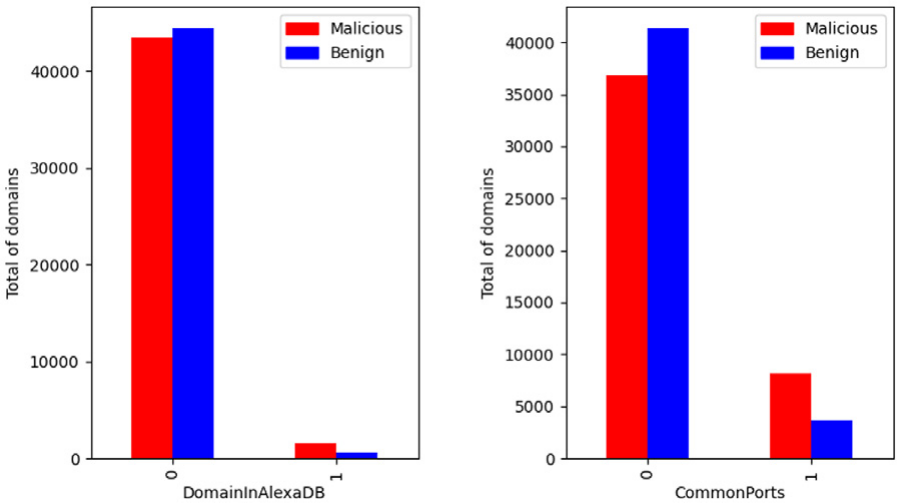
DomainInAlexaDB and CommonPorts by class.

A simple plot between the DomainInAlexaDB and CommonPorts, illustrated in Fig. 4, reveals that the distribution of features according to the class is uniform and similar. The first plot shows that there are many domains in the dataset that are not included in the Alexa DB [6], regardless of class. The second shows that for a significant part of the domains there are no common active ports.

**Fig. 5:**
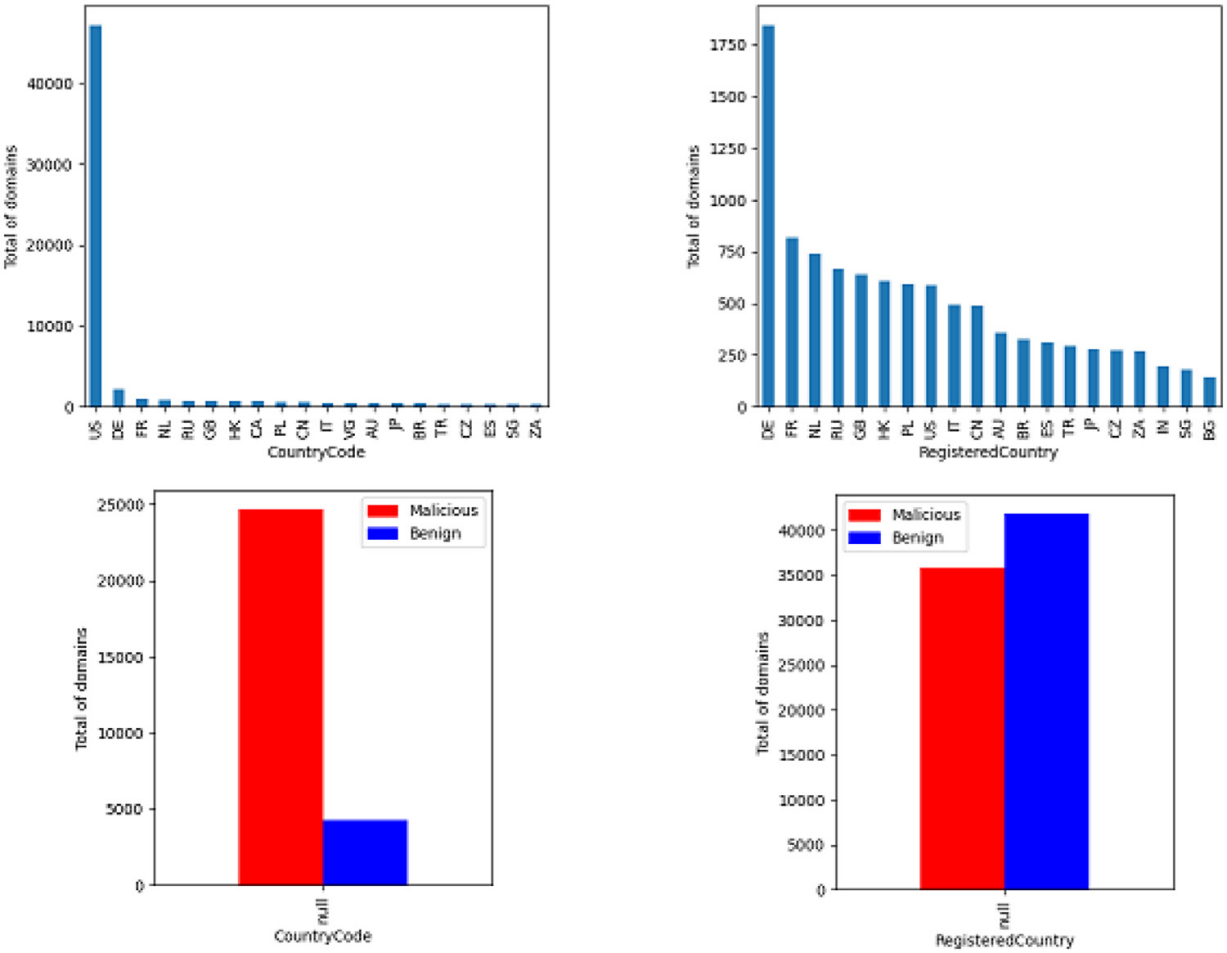
Geographic information and null count by class.

The CountryCode and RegisteredCountry features reveal geographic information about the domain or the IP associated with the domain. These features are illustrated in Fig. 5 is illustrated. With regard to the CountryCode, it appears that most IP are active in the USA and that they are mostly malicious. This has to do with the data source implying that malicious domains are largely associated with servers hosted in the USA (they may be sinkhole DNS servers). The RegisteredCountry focus on the domain information (collected using the WHOIS database). It shows a greater geographical dispersion and distributed among the classes under analysis. This analysis does not consider the null values and was used the top 20 country codes for presentation purposes.

**Fig. 6:**
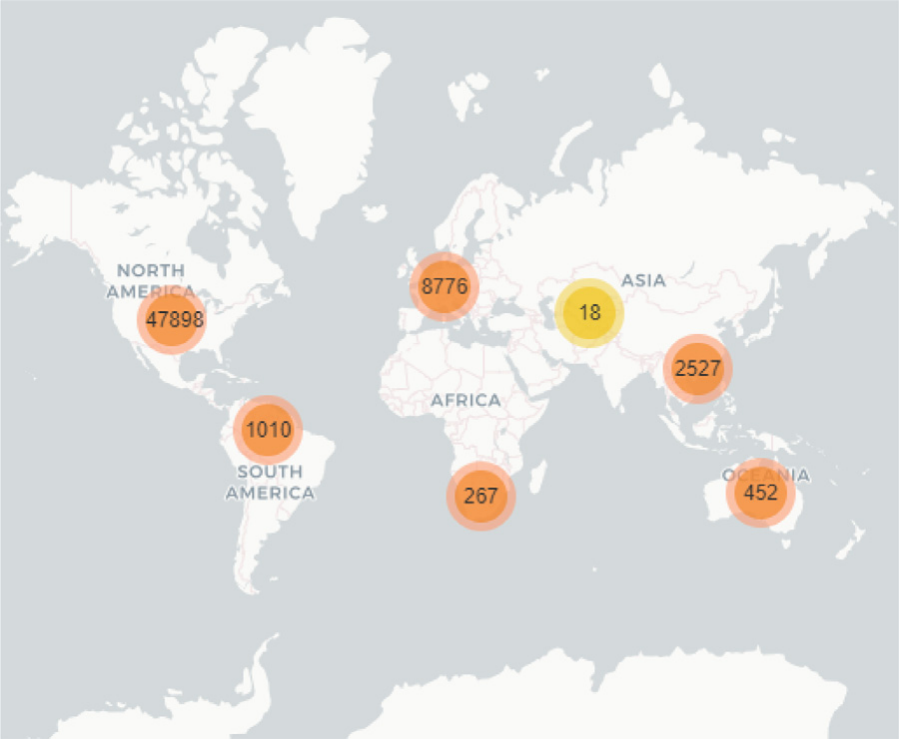
Country Code geographic distribution in a world map.

**Fig. 7:**
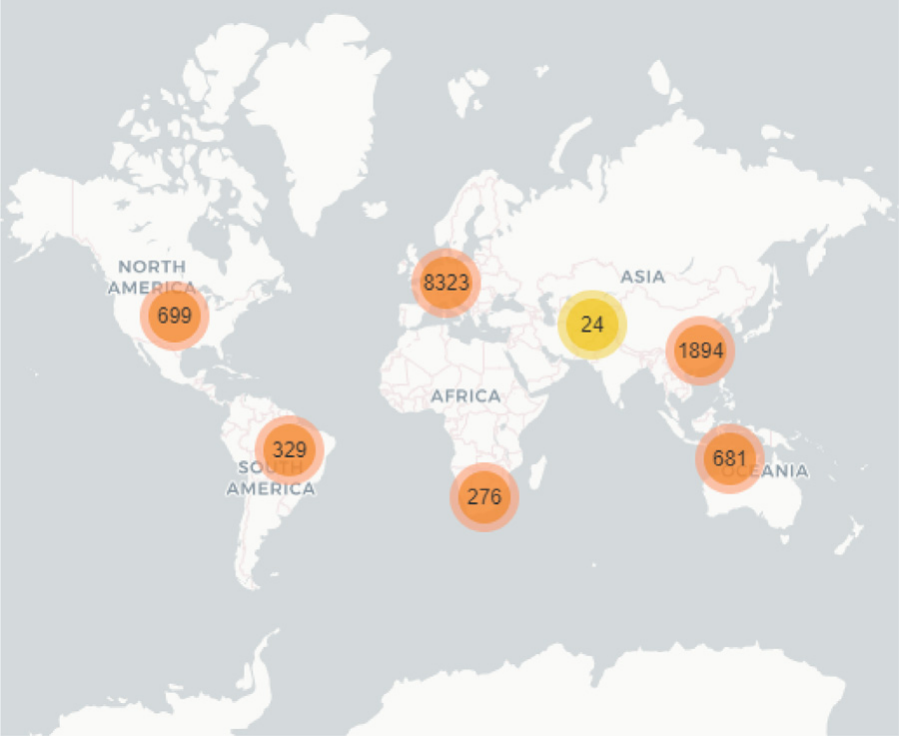
Registered Country geographic distribution in a world map.

Figures 6 and 7 presented a different view for the CountryCode and RegisteredCountry labels respectively.

**Fig. 8:**
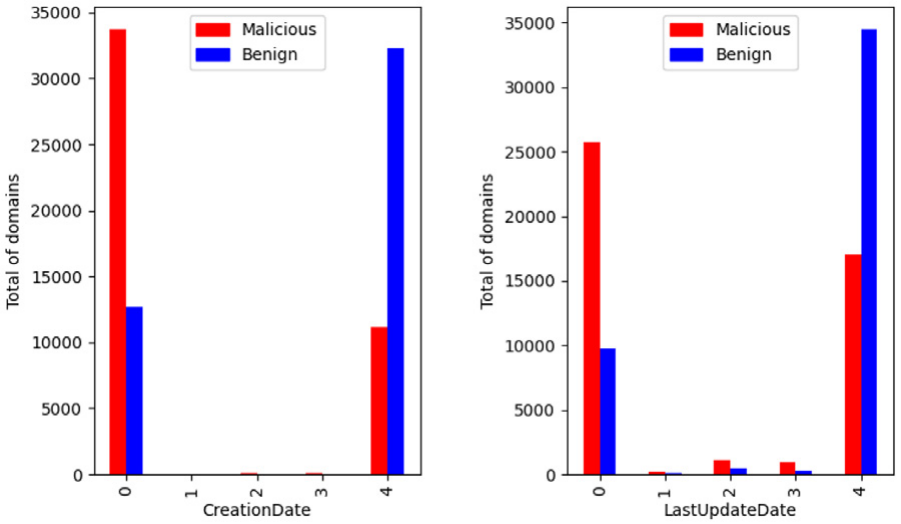
Domain creation and last update date per class.

The domain registration/creation date and last update date uses an enumeration to compress the range of dates. In Fig. 8 these features are illustrated grouped by the class label. This analysis is important to understand if malicious domains are created and changed more frequently than non-malicious domains (older and more stable, particularly for well-known domains).

**Fig. 9:**
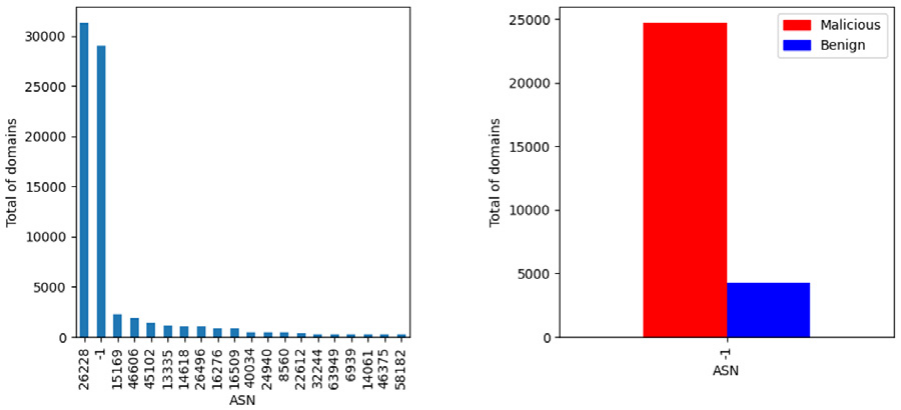
ASN data distribution per class.

The Autonomous System Number (ASN) distribution is illustrated in Fig. 9. The minus one value means that there is no ASN information for the domain. From the figures is also possible to observe the not found (negative) values regarding the class label. It is important to refer that the ASN information is related with the existence of an IP address associated with the domain, so it is not strange at all that the output of this analysis is very similar to IP analysis previously presented. Once more it is possible to verify the influence of ASN 26228. This is not due to outliers but it seems to result from the data origin.

**Fig. 10:**
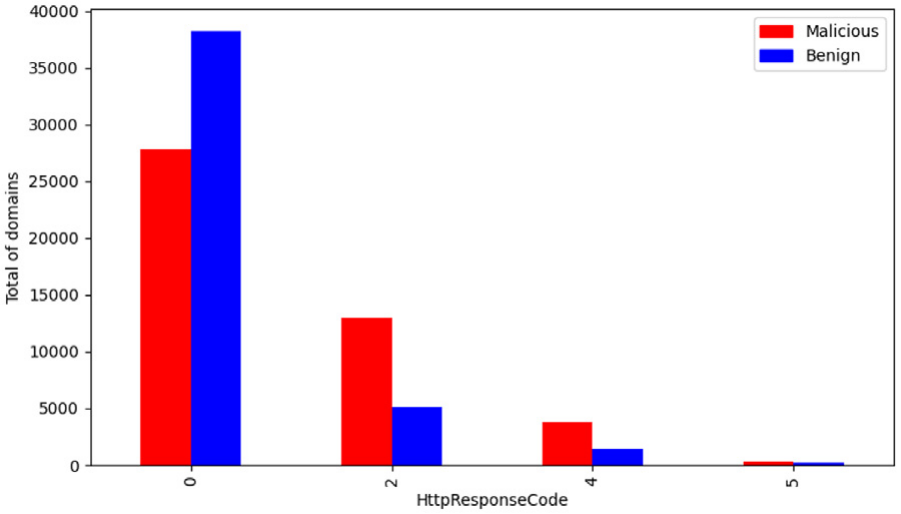
HTTP / HTTPS response by class.

The Hypertext Transfer Protocol (HTTP) / Hypertext Transfer Protocol Secure (HTTPS) response code enumeration is described in Table 2. In Fig. 10 it is possible to see the distribution of the response code by the class label.

**Fig. 11:**
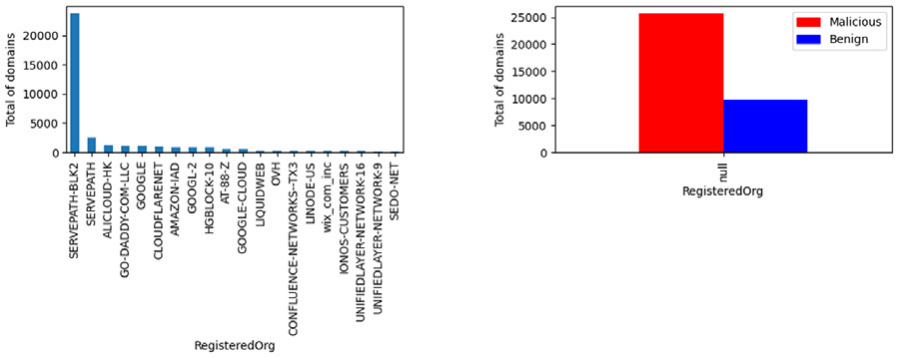
Registered Organization label distribution.

The registered organization feature results from querying the WHOIS service. In Fig. 11 it illustrated the top 20 organizations in the presented dataset. Typically, a non-malicious organization does not hide this information when registering the domain. On the other hand, malicious actors tend not to reveal this information or to tamper with it. In the figure is presented the relationship between the “null” values (i.e. when the information is not available) per class.

**Fig. 12:**
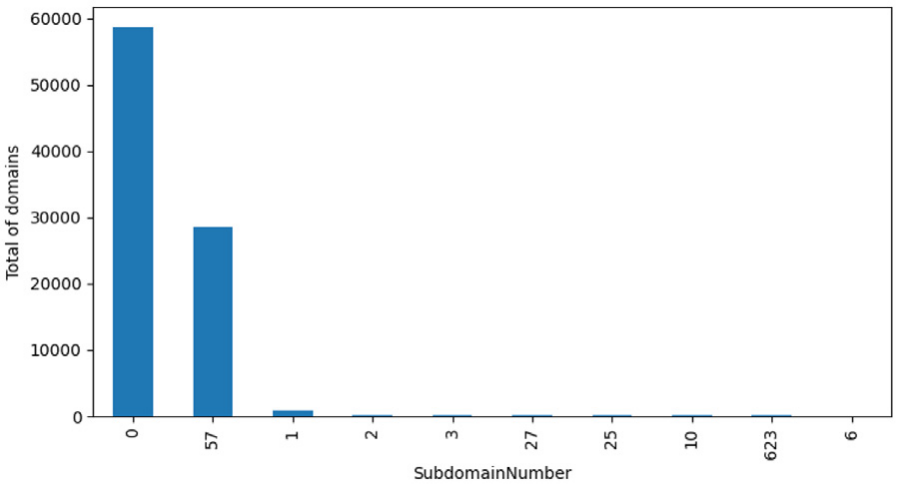
Sub domains label distribution.

The subdomain feature (Fig. 12) allows checking if a given domain has subdomains registered. The rationale behind this parameter is to follow. It is normal for a real organization to have multiple sub domains associated with the domain. On the contrary, a malicious domain will not normally have many associated sub domains. The figure shows the top 10 most frequent sub domain count and the number of domains that fit in each count. From the illustration we observer that more then 55 thousand domains do not have sub domains associated. From the dataset it is also possible to improve the analysis. For example, it is possible to do a cross-table between the number of sub domains and their class.

**Fig. 13:**
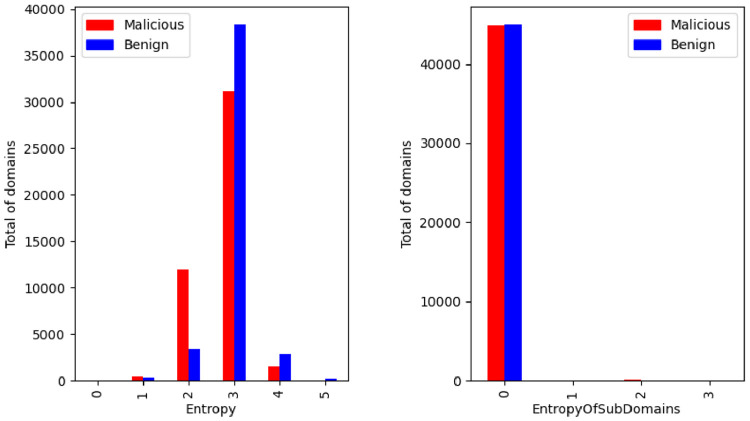
Entropy of domain and mean entropy of sub domains by class.

Another features present in the dataset are the domain entropy and the mean entropy value obtained from the entropy of each sub domain. The result was rounded to integer values and the distribution by the class label is illustrated in the Fig. 13.

**Fig. 14:**
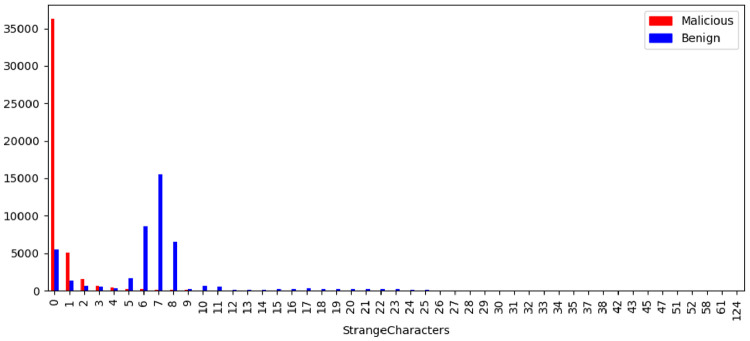
Strange Characters label distribution by class.

Fig. 14 illustrates the strange characters feature per class. The domain name is part of an organization identity, so it is expected that the name will be chosen in order to be easily used and memorized. The existence of strange characters contradicts these logic and can serve as an indicator of the existence of domains for malicious purposes.

**Fig. 15:**
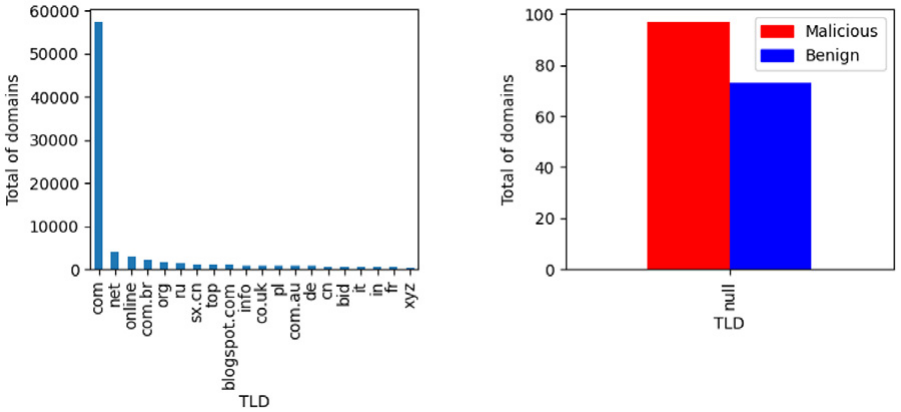
TLD label distribution and null values per class.

The Top Level Domain (TLD) feature per class is illustrated in Fig. 15. For presentation purposes only the top 20 TLD are presented. The null values per class are illustrated in the right-side of the figure.

**Fig. 16:**
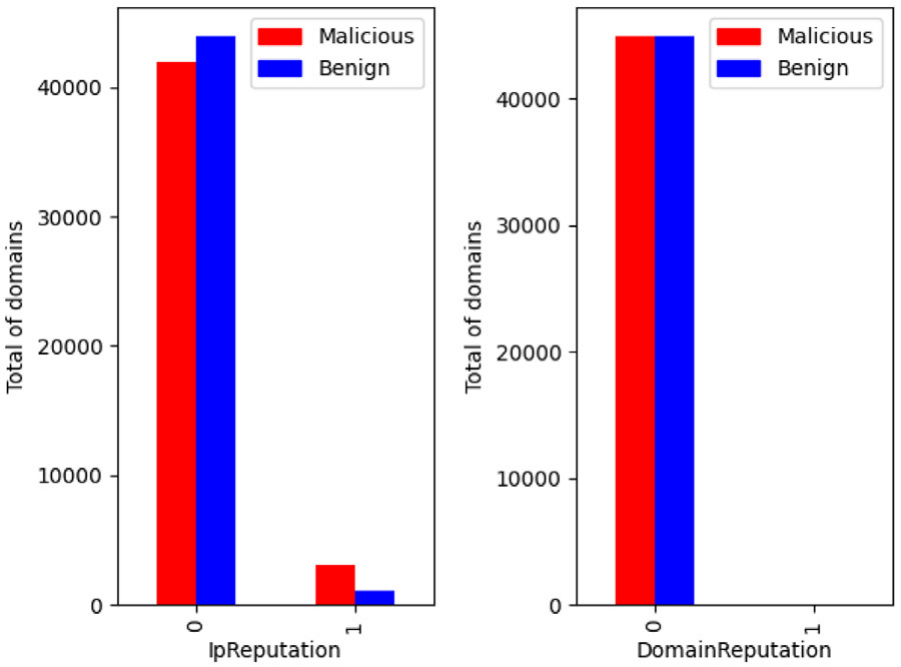
IP and domain reputation labels distribution per class.

The IP and domain reputation feature per class is illustrated in the Fig. 16. Both parameters result from the classification made by third parties and are widely used in the area of cybersecurity to identify possible IP and malicious domains. Their existence in the dataset is justified and it will be interesting to ascertain the weight they will have for the classification process.

**Fig. 17:**
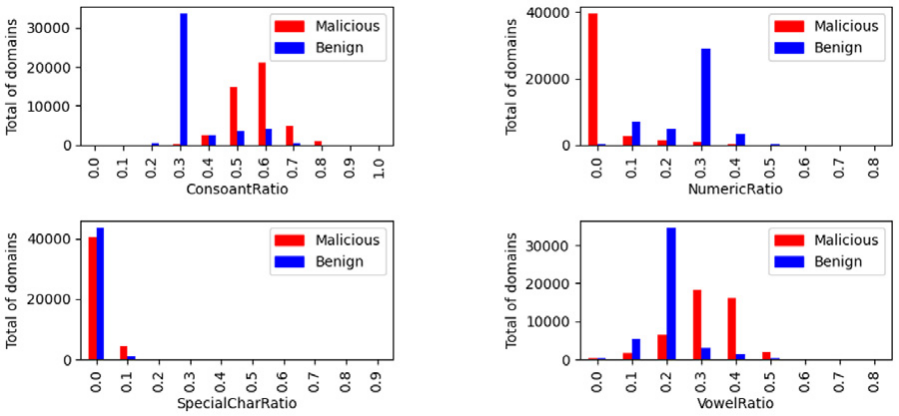
Ratios distribution by class.

**Fig. 18:**
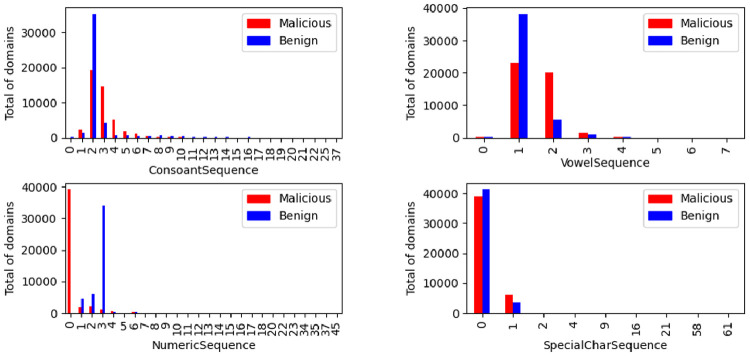
Sequence distribution by class.

The distribution of the ratio of vowels, consonants, numeric and special characters in the domain are illustrated in Fig. 17. The same representation was made for the sequences illustrated in Fig. 18. The combined analysis between these parameters is interesting, as it is expected that a non-malicious domain name will be created in order to be easily memorized and used.

**Fig. 19:**
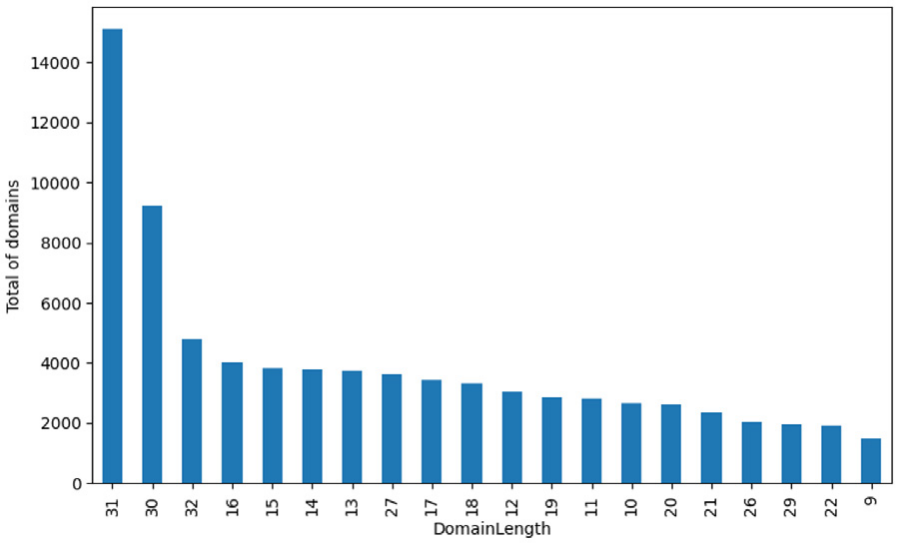
Domain length label distribution.

The domain length per class is illustrated in Fig. 19. Once more, for presentation purposes, a frequency chart with the 20 most common domain sizes is presented.

## 2 Experimental Design, Materials and Methods

**Fig. 20:**
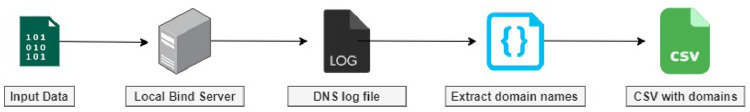
DNS requests and extraction process.

To create the dataset we started from lists of already classified malicious and non-malicious domains (data sources). All started with a simple domain name and for each domain a DNS query was performed (Fig. 20). The results were logged and then processed using Python. The Python processed over 45000 non-malicious and 45000 malicious domains. Python modules allow, from the domain name, to obtain the features presented in Table 1.

In a real scenario the data is obtained from the DNS server and not from structured public available files. To mimics it we replicated the DNS queries. To do so we configured a Local DNS Bind server [7] allowing to collect the DNS queries and responses. These response was outputted to a log file that was parsed and a CSV file with the domains names was finally create. The CSV with the domains is then used to extract the dataset features.

Presenting the details of the Python modules is not the objective of this article, although we leave an example of a Python module that allows to obtain the IP address for a given domain. This Code Snippet 1 is part of the information gathering (data enrichment) process and it makes use of the socket native python library to retrieve the IP from a domain name. To maximize the results some of the domains were concatenated with the the “www” sub domain prefix.

**Code Snippet 1:**
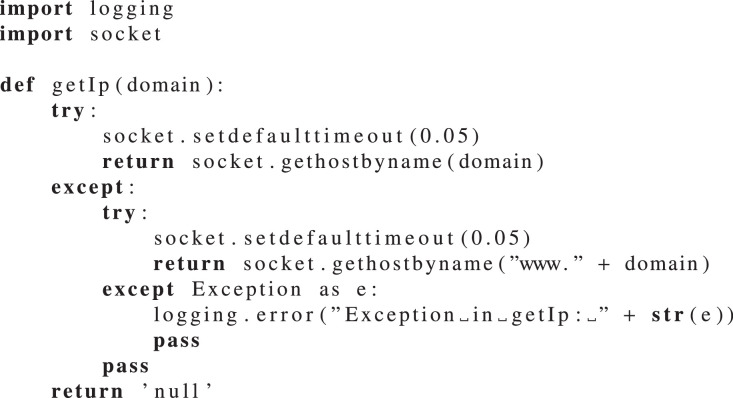
Function to get the IP for a given domain.

The full Python code used to create the dataset is available in [8]. The structure of the code is based on the main Python file that calls functions in different modules and makes use of a utilities and data stored in different directories. The structure is as follow:

• main_create_datasets.py - The main file to create the dataset and order all the steps starting in the data collection to information gathering.
• data/ - Inside the folder there are two sub folders (input and output). The logs collected must be inside the input folder categorized by the DNS record type and by the class. The output sub folder will be the path for the final dataset.
• lib/ - Contains the functions and modules to support the information gathering process.
• utils/ - Contains utility functions in Python Scripts, such as the constants to be used in run-time. It contains a database sub folder with the AlexaDB [6] and GeoIp [9] databases inside. The data tools sub folder contains the functions to the collection of the domains from the logs files and for the information gathering.

The third-party libraries/data used to collect information from a domain are:

• AlexaDB [6] - Alexa top 1 million sites.
• geoip2 [9] - GeoIp Database.
• pydnsbl [10] - Anti-spam blacklists domain or IP checker.
• sublist3r [11] - Python package to enumerate sub domains of a given domain using OSINT. The engines used were the PassiveDNS and Bing.
• tld [12] - Python package to extract the TLD.
• IPWhois [13] - Python package to retrieve information from WHOIS.

**Fig. 21:**
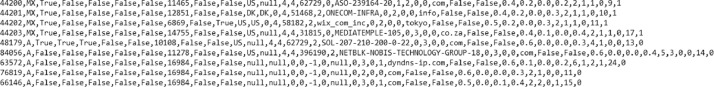
Dataset result snippet.

The result of the information gathering process is stored in a CSV file. A small excerpt of the result is illustrated in Fig. 21. To protect the identity behind each domain, the domain column was anonymized using the Label Encoder from SkLearn framework [14] with a value between 0 and n_classes−1. The dataset is publicly available at [8].

## Ethics Statement

The work did not involve any human subject or animal experiments. The values of the IP column which are considered personal data under the General Data Protection Regulation (GDPR) rules were anonymized.

## CRediT authorship contribution statement

**Cláudio Marques:** Investigation, Software, Data curation, Writing – original draft, Visualization. **Silvestre Malta:** Formal analysis, Writing – review & editing. **João Paulo Magalhães:** Conceptualization, Methodology, Formal analysis, Writing – review & editing.

## Declaration of Competing Interest

The authors declare that they have no known competing financial interests or personal relationships which have, or could be perceived to have, influenced the work reported in this article.

## References

[1] Rapid7 Labs, 2020, URL: https://opendata.rapid7.com/sonar.fdns_v2.

[2] SANS Internet Storm Center, 2020, URL: http://web.archive.org/web/20200503151842/https://www.dshield.org/feeds/suspiciousdomains_Low.txt.

[3] Kaur M.S.M.S.S. (2019).

[4] Berkay Celik Z., Oktug S. (2013). 2013 IEEE Symposium on Computers and Communications (ISCC).

[5] Yadav S., Reddy A.K.K., Reddy A.L.N., Ranjan S. (2012). Detecting algorithmically generated domain-flux attacks with DNS traffic analysis. IEEE/ACM Trans. Netw..

[6] Amazon, AWS Alexa Top 1M, 2020, URL: http://s3.amazonaws.com/alexa-static/top-1m.csv.zip.

[7] Bind 9 - ISC, 2021, URL: https://www.isc.org/bind/.

[8] C. Marques, Dataset Creator, 2021, URL: https://github.com/claudioti/dataset-creator.

[9] MaxMind, GeoIP2 Databases, 2020, URL: https://www.maxmind.com/en/geoip2-databases.

[10] D. Ippolitov, pydnsbl, 2020, URL: https://pypi.org/project/pydnsbl/.

[11] Sublist3r, 2020, URL: https://github.com/aboul3la/Sublist3r.

[12] A. Barseghyan, TLD, 2020, URL: https://pypi.org/project/tld/.

[13] P. Hane, IPWhois, 2020, URL: https://pypi.org/project/ipwhois/.

[14] Scikit Learn, 2020, URL: https://scikit-learn.org/stable/.

